# Extracellular Matrix Molecules Facilitating Vascular Biointegration

**DOI:** 10.3390/jfb3030569

**Published:** 2012-08-14

**Authors:** Steven G. Wise, Anna Waterhouse, Praveesuda Michael, Martin K.C. Ng

**Affiliations:** 1 The Heart Research Institute, Eliza Street, Newtown, NSW 2042, Australia; Email: wises@hri.org.au (S.G.W.); michaelj@hri.org.au (P.M.); 2 Wyss Institute for Biologically Inspired Engineering at Harvard, Boston, MA 02115, USA; Email: anna.waterhouse@wyss.harvard.edu

**Keywords:** vascular, biointegration, tropoelastin, fibrillin-1, perlecan, fibulin-5

## Abstract

All vascular implants, including stents, heart valves and graft materials exhibit suboptimal biocompatibility that significantly reduces their clinical efficacy. A range of biomolecules in the subendothelial space have been shown to play critical roles in local regulation of thrombosis, endothelial growth and smooth muscle cell proliferation, making these attractive candidates for modulation of vascular device biointegration. However, classically used biomaterial coatings, such as fibronectin and laminin, modulate only one of these components; enhancing endothelial cell attachment, but also activating platelets and triggering thrombosis. This review examines a subset of extracellular matrix molecules that have demonstrated multi-faceted vascular compatibility and accordingly are promising candidates to improve the biointegration of vascular biomaterials.

## 1. Introduction

With advances in technology, implantable cardiovascular devices such as grafts, stents, pacemakers and heart valves are playing an increasingly important role in modern cardiovascular medicine. Despite this, biocompatible and clinically effective synthetic materials for cardiovascular devices are currently lacking. Current artificial grafts are predominantly made of either Dacron or expanded polytetrafluoroethylene (ePTFE). These artificial materials lack sufficient elasticity, have poor interactions with vascular cells, are highly thrombogenic and induce chronic inflammation [1].Re-endothelialization of graft surfaces is retarded by the highly hydrophobic surfaces and smooth muscle cells proliferate in response to the inflammatory cascade. Accordingly, current synthetic conduit materials fail uniformly in the treatment of peripheral vascular disease within 5 years due to thrombosis and uncontrolled cellular infiltration [2,3]. There remains a strong demand for novel grafts due to these and other shortcomings. 

The problem of innate incompatibility extends beyond graft materials to other vascular devices such as heart valves, pacemaker components and stents. These devices are primarily made of metal alloys, which are highly thrombogenic and induce inflammation at the site of implantation. The thrombogenicity of metal alloys are highlighted by the need for lifelong anticoagulation in metal heart valve recipients [4] and by the worrying propensity for coronary stents to thrombose. While the incidence of stent thrombosis for bare metal platforms is relatively low when used with antiplatelet therapy, such an event is catastrophic, associated with mortality rates of 25% [5]. Drug eluting stents (DES) releasing anti-proliferative agents are highly effective in inhibiting neointimal hyperplasia but also profoundly delay healing and re-endothelialization at the stent deployment site [6]. Consequently, the safety of DES has come into question [7]. DES are not only susceptible to early thrombotic events like BMS, but are also prone to both late (30 days–1 year) and very late (>1 year) stent thrombosis [8]. In stable patients with single vessel disease, late stent thrombosis occurs at a constant rate of 0.6% per year with no indication that this abates in the long term [9]. However, in real world studies incorporating acute coronary syndromes and complex interventions, even higher rates have been reported (0.9–3%/year) [10]. Additionally, DES polymer instability has been associated with chronic inflammatory and hypersensitivity reactions [11]. 

**Figure 1:**
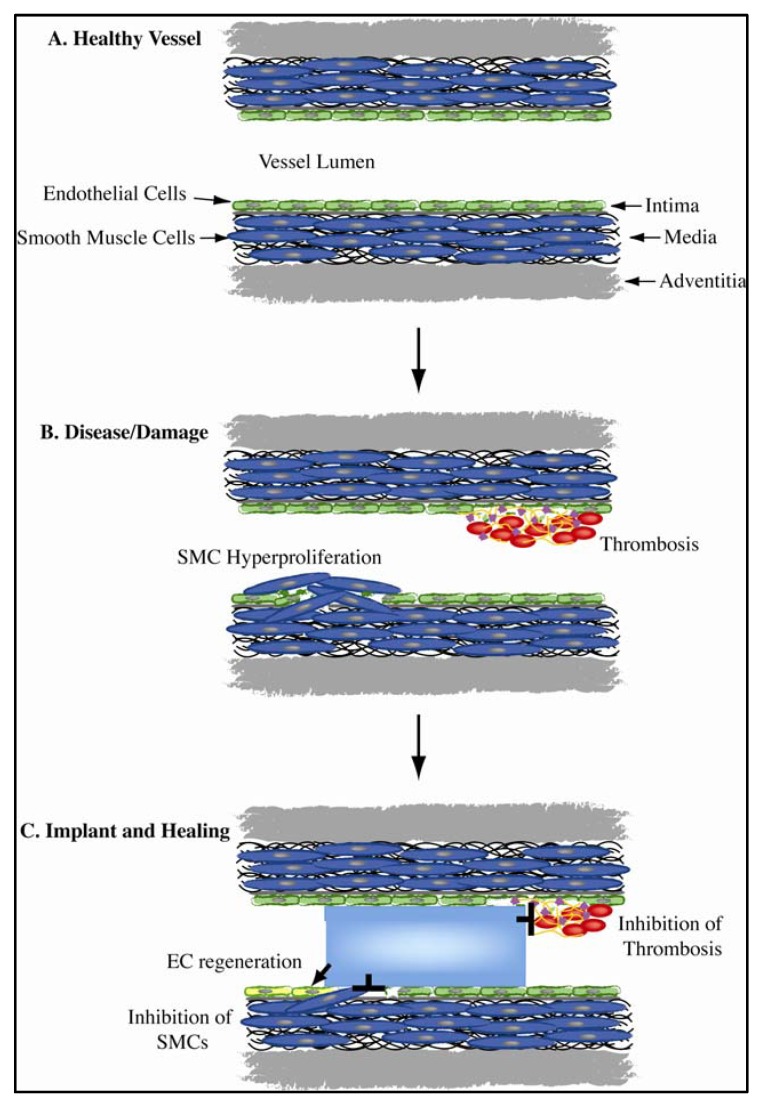
The multiple facets of vascular biointegration: (**a**) A healthy vessel is characterized by a complete monolayer of endothelial cells (ECs), quiescent smooth muscle cells (SMCs) and hemostasis; (**b**) In disease, the endothelium is compromised, SMCs hyper-proliferate and the clotting cascade is triggered; (**c**) An ideal implanted device will simultaneously enhance EC regrowth, block SMC proliferation and inhibit thrombus formation.

As a consequence of the lack of biocompatible synthetic materials, a key objective of vascular bioengineering is the development of new biomaterials with appropriate biological properties that mimic that of human arteries. The commonality of vascular implants is the requirement to simultaneously modulate multiple biological processes (Figure 1). Injured and diseased vasculature routinely has damaged and compromised endothelium, while the inflammatory response triggers hyper proliferation of smooth muscle cells (SMCs). In this environment, clotting cascade factors are activated and thrombosis is easily initiated. An optimal medical device will encourage rapid regeneration of the endothelium; inhibit SMC proliferation and migration while having high blood compatibility.

### 1.1. Vascular Biomimicry

Classically, materials exhibiting biocompatibility perform their function without eliciting an undue host response or resulting in adverse clinical outcomes [12]. However, this paradigm has shifted recently beyond inertness, requiring biocompatible materials to facilitate beneficial cell and tissue interactions. In this context, the design of new vascular devices should aim to promote rapid and appropriate healing. A balance needs to be reached between low thrombogenicity, rapid re-endothelialization and arresting the infiltration and proliferation of SMC’s. An ideal vascular implant would be non-thrombogenic even when in contact with the circulation and in the absence of platelet inhibition. 

Biomimetics or imitation of natural methods and processes has been identified as a means of significantly improving the biocompatibility of vascular materials. Biomimicry strategies in the context of vascular devices have been diverse including: whole organ tissue scaffolds [13], host derived vascular grafts [14] and biomolecule coatings with cell interactive characteristics [15]. Previous attempts to develop bioactive coatings for vascular implants have been limited by overly simplistic approaches, often focusing on modulating one facet of biocompatibility (e.g., endothelialization alone) at the expense of others. For example, despite having favorable endothelial cell interactions, collagen, fibronectin and laminin are thrombogenic [16,17,18]. Additionally, vitronectin, collagen (III and IV) and fibronectin promote smooth muscle cell migration [19,20]. Peptides mimicking cell-binding motifs in fibronectin (RGD) and laminin (YIGSR) are also widely used, but suffer the same shortcomings as their parent proteins, failing to achieve multi-faceted effect [21,22]. Similarly, phosphorylcholine (PC), based on a naturally occurring cell membrane lipid, was observed *in vitro* to be non-thrombogenic when used as a stent coating [23]. *In vivo*, PC failed to encourage endothelialization and ultimately had no effect on the rate of stent thrombosis [24]. Overall, a successful biomimicry approach will extend beyond emulation of a single desirable vascular property and more broadly seek to address a balance between favorable cell interactions and reducing thrombogenicity.

### 1.2. Candidate Biomolecules from the Vessel Wall

A range of biomolecules involved in the regulation of thrombosis and cell signaling are found in the vessel wall. The extracellular matrix (ECM), a complex collection of diverse proteins that form an organized network to which cells adhere [25], comprises the underlying structural component of vessels. The ECM modulates cell behavior [26] and signaling [27] and is responsible for the structural support of tissues. The ECM of the vasculature is primarily composed of collagens for structure, elastin for recoil, various glycoproteins (including microfibrils) for adhesion and proteoglycans and glycosaminoglycans, which provide many diverse functions.

Elastic fibers are major ECM assemblies that provide elasticity and resilience. These contain primarily elastin, in combination with fibrillin-1 and co-localized with fibulin-5 [28]. Both fibrillin-1 and fibulin-5 enhance endothelial cell stability and inhibit SMC migration [29]. The elastin rich internal elastic lamina is also a notable structural feature of large arteries, critical in preventing the infiltration of SMCs. The basement membrane, recognized as an essential mediator of cell homeostasis, provides structural support to endothelial cells and regulates their behavior. This sheet-like structure contains numerous proteins including type IV collagen, laminins, small glycoproteins such as nidogen/entactin, and heparin sulphate proteoglycans such as perlecan [30]. Here, perlecan is of particular interest as it is implicated in cell binding and proliferation, growth factor regulation [31] and the inhibition of thrombosis [32].

**Figure 2:**
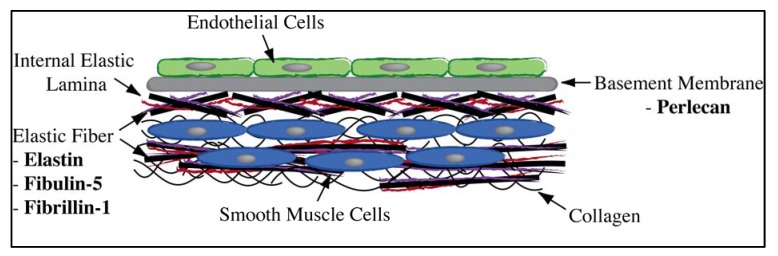
Candidate biomolecules and their relative locations in the vessel wall, highlighting their proximity to ECs, SMCs and the vessel lumen.

Despite the wide variety of proteins present in the extracellular matrix and basement membrane, many have features that are not desirable for use as a biomaterial coating, as discussed for collagen and fibronectin above. The ideal candidate biomolecule would enhance healing and endothelialization, reduce smooth muscle cell hyperproliferation caused by inflammation and be non-thrombogenic. Overall, there is a subset of biologically relevant candidates with the potential to improve implant biointegration. These include elastin, fibrillin-1, fibulin-5 and perlecan (Figure 2), which have all been shown to play important development or regulatory roles in the vasculature, while simultaneously influencing re-endothelialization, inhibiting smooth muscle cells and deterring thrombosis (Figure 2). We discuss these four promising candidates in further detail below.

## 2. Candidate Biomolecules

### 2.1. Fibrillin-1

Microfibrils are an important component of elastic fibers, providing both mechanical and cell interactive properties. Across tissue types it is proposed that microfibrils are present at the beginning of elastogenesis and act as a scaffold for tropoelastin, the soluble precursor of elastin, deposition and assembly [33]. They are adjacent to tropoelastin producing cells and parallel to the developing fiber [34]. While the specifics of their role in assembly are not known, microfibrils have been described as having a bead-on-a-string appearance [35], while there is also evidence of small filaments that span between the adjacent microfibrils [36] suggesting some lateral assembly interaction. Microfibrils are primarily composed of glycoproteins from the fibrillin family and microfibril-associated glycoprotein (MAGP)-1 and -2. Fibrillin-1 and -2 have so far been implicated as key microfibrillar components, both are found extracellularly, with different but coincident patterns of expression. Of the three fibrillins so far identified, fibrillin-1 has particular importance to the cardiovascular system, while fibrillin-2 and -3 are highly expressed in lung tissue and cartilage, though with differential expression patterns [37].

Fibrillin-1 is a 350 kDa cysteine-rich glycoprotein which has been shown to be particularly important in elastic fiber development [38] and wound repair [39]. It contains 47 epidermal growth factor (EGF) domains, of which 43 are calcium binding (cbEGF)-like domains, and 7 eight-cysteine-containing TB motifs. It is currently theorized that tropoelastin deposition on the microfibril is directed toward the inter-bead region and corresponding concentrations of fibrillin-1 [40], implying an important signaling role in elastic fiber assembly. It interacts strongly with cells via a canonical arginine-glycine-aspartic acid (RGD) motif, as well as with other ECM proteins including decorin, versican and tropoelastin [41]. 

The importance of fibrillin-1 to the vasculature is demonstrated by autosomal dominant mutations in the fibrillin-1 gene known to cause Marfan Syndrome (MFS) [42]. This disorder has significant skeletal and pulmonary complications, but manifests in the vasculature as aortic aneurysm and weakened heart valves, linked to reduced microfibrillar integrity and vascular mechanics. Marfan patients also exhibit EC dysfunction implying a role for fibrillin-1 in anchoring and signaling to this cell type. Fibrillin-1 knockout mice, used to model MFS also demonstrate progressive fragmentation of their aortic elastic lamellae, fragmentation of microfibrils in other tissues and EC detachment, providing further evidence for its importance [43]. This disease state also points to an important biological role for fibrillin-1 in regulating the bioavailability of transforming growth factor beta (TGF-β), a critically import cell regulatory molecule [44].

Studying the biological effects of fibrillin-1 *in vitro* has benefited from a molecular biology approach, recombinantly producing discreet domain segments. Details of this approach and the fragment nomenclature have been previously published [45]. Fibrillin-1 constructs spanning the full-length protein have assisted in elucidating important cell interactive regions, such as the RGD present in PF8/PF9 [45], as well as the contribution of surrounding stabilizing domains [46].

Fibrillin-1 binds to a range of cell types including endothelial cells, smooth muscle cells and fibroblasts. This interaction is mediated by α_v_β_3_ and α_5_β_1 _integrins binding to the single RGD motif found in fibrillin-1 [47]. Polar residues surround the RGD sequence, making it highly likely to be solvent exposed and accessible for binding interactions. Fibrillin-1 fragments containing the RGD sequence, such as PF8 and PF9 (and constructs such as PF14 which combine PF8 and PF9) are suggested to be responsible for promoting endothelial cell proliferation via this mechanism. However, the binding interaction is complex, requiring contributions from upstream cbEGF domains to position the RGD sequence to achieve full adhesion and signaling [46]. This is exemplified in the differential binding behavior seen between PF9 (RGD only) and PF14 constructs (RGD and surrounding domains), with only PF14 actively signaling into human umbilical vein endothelial cells [48]. The enhanced signaling mediated by PF14 was directly shown to enhance endothelial cell proliferation and migration. These findings are consistent with data from fibrillin-1 null mice, which show thinner and disorganized elastic lamellae leading to a detached endothelial lining [49]. Together these data support an important role for fibrillin-1 in the regulation of endothelial cell binding and signaling as one facet of a wider biological role relevant to arterial morphogenesis and physiology [49]. 

Importantly, fibrillin-1 fragments have also been shown to signal smooth muscle cells and inhibit their migration. Specifically, PF8 and PF9 constructs have demonstrated dramatically lower migration rates in a Boyden chamber assay in comparison to fibronectin [29]. Fibrillin-1 also appears to regulate the production of matrix metalloproteinase (MMP) and directly impact on the health and phenotype of vascular SMCs. In Fibrillin-1 deficient mice, SMCs are less well adhered, adopt a synthetic and proliferative phenotype and have up-regulated MMP9, leading to fragmentation of elastic lamellae and an enhanced inflammatory response [50]. Similar findings are observed in human MFS patients [51], demonstrating the importance of fibrillin-1 in SMC signaling.

Direct assessment of the blood compatibility of fibrillin-1 (or any fibrillin) has not yet been carried out, but studies of the thrombogenicity of vessel wall components infer their relative compatibility. Decellularized elastic fibers, containing microfibrils and an elastin core, have been shown to bind fewer platelets than collagen or isolated basement membrane [52]. While elastin in particular is highly compatible with blood (discussed in more detail below), microfibrillar components were shown to cause much lower platelet activation than collagen [53]. It has also been assumed that proteins containing an RGD sequence would strongly bind platelets and induce thrombus formation, as is the case for collagen and fibronectin. However, recent work functionalizing materials with RGD inspired peptides and demonstrating improved blood compatibility [54], challenge this model and a direct assessment of the effect of fibrillin-1 on coagulation is needed. Initial work showing the potential benefits of modifying the surface of a biomaterial with fibrillin-1 has been carried out using a polyurethane scaffold. Immobilized PF9 was shown to significantly enhance the binding and spreading of fibroblasts to the material surface and was notably superior to an RGD peptide originating from the sequence of fibronectin [55]. This demonstration supports the idea that not all RGD sites are equivalent.

### 2.2. Fibulin-5

The fibulins are a family of secreted glycoproteins associated with basement membranes, elastic fibers, and other matrices and are expressed in a variety of tissues. There have been seven fibulins identified, divided into class I and class II based on length and domain structures. Class II fibulins, called “short” fibulins, include fibulin-3, -4, -5 and -7. Of these, fibulin-5 is the most prominently expressed in the vasculature, co-localizing with all layers of elastic lamina and present on the surface of elastic fibers [56].

The importance of fibulin-5 (also called EVEC or DANCE) to normal elastogenesis was first demonstrated by fibulin-5^−/−^ mice that show massively disorganized elastic fibers, resulting in aberrant skin, vascular and lung phenotypes [57]. Given that fibulin-5 is known to interact with the elastic fiber molecules, tropoelastin and fibrillin-1 [58] it was proposed to play an important role in fiber assembly, tethering the developing structure to cell surfaces. However, mice expressing mutant fibulin-5 with a disrupted RGD sequence display normal fiber formation, indicating that their role in elastogenesis requires further investigation [56] and is not limited to cellular interactions. 

From a vascular perspective, fibulin-5 is of particular interest as it is abundantly expressed in developing arteries. In adult blood vessels, expression is at low levels, however, the expression can be restarted in response to vascular injury and is enhanced in atherosclerotic plaques [59]. Following vascular injury, fibulin-5^−/−^ mice present excessive SMC proliferation and migration and are more susceptible to thrombus formation. Vessels were found to be less elastic and remodeling impaired, while highlighting an unexpected role for fibulin-5 in SMC regulation [60]. Fibulin-5 also cooperates with fibulin-2 in the formation of the internal elastic lamina. Mice lacking both proteins show severely disorganized lamellae, thinner vessel walls and display higher rates of thrombosis following injury [61].

Fibulin-5 is the only fibulin to contain a conserved RGD motif, known to mediate binding to cell surface integrin receptors present on both endothelial cells and vascular smooth muscle cells [62]. For EC’s, binding is mediated by integrins including α_v_β_3_ and α_v_β_5_, while fibulin-5 also interacts strongly with TGF-βανδ vascular endothelial growth factor (VEGF), implicating a role in regulating angiogenesis and in modulation of endothelial cell function [63]. *In vitro*, over-expression of fibulin-5 in primary endothelial cells enhanced their attachment, but impaired proliferation. Dual expression with VEGF in the same cell type improved cell proliferation while maintaining a high level of attachment [64]. The importance of fibulin-5 in the protection of ECs is highlighted in hypoxic conditions, where fibulin-5 gene expression is increased and the rate of apoptosis in fibulin-5 knockdown cells is greater [65]. Surfaces coated with fibulin-5 exhibit strong integrin-mediated EC attachment in static and flow conditions at a level comparable to fibrillin-1 and the cells form stable, functional monolayers [29].

The interaction of fibulin-5 with smooth muscle cells occurs via a different subset of integrins and has been shown to have an inhibitory effect. Vascular SMCs from fibulin-5^−/−^ mice have enhanced proliferative and migratory behavior compared to wild-type cells, a response inhibited by overexpression of fibulin-5 [60]. This inhibition occurred in the absence of the α_v_β_3 _integrin demonstrating the presence of additional mediators. The attachment of primary human aortic SMCs to recombinant fibulin-5 was subsequently shown to be through α_5_β_1_ and α_4_β_1_ integrins. However, SMCs in this study were poorly attached and spread and showed inferior migration and proliferation in comparison to fibronectin [58]. Migration of SMCs to a confluent layer of ECs grown on fibulin-5 was also reduced, in further support of the inhibitory effect of fibulin-5 on SMCs [29].

The blood interactions of fibulin-5 have not been studied, though the increased thrombosis observed in fibulin-5/fibulin-2 double knockout mice infers a role in maintaining hemostasis. As with fibrillin-1, the presence of fibulin-5 in association with elastic fibers is also encouraging given the reduced interactions these have with platelets. As discussed above, the presence of an RGD is not necessarily a barrier to blood compatibility.

### 2.3. Perlecan

Perlecan is a large (467 kDa) multidomain heparan sulfate proteoglycan (HSPG) that is expressed in most ECM and basement membranes and is essential for the assembly and maintenance of a functional basement membrane. It is the major extracellular HPSG present in blood vessels and consists of a core made up of five distinct domains that each interact with molecules involved in cell proliferation, lipoprotein uptake and cell adhesion [66]. In vertebrates, perlecan functions in a diverse range of developmental and biological processes, including the regulation of wound healing and modulation of vascular biology [31]. It has been found to co-localize with fibrillin-1 and elastin in blood vessels [67] and to play an important regulatory role in sequestering a host of growth factors and cell signaling molecules [68]. Other heparan sulfate proteoglycans include syndecans and glypicans, however these are membrane bound [69]. Secreted HSPGs, agrin and collagen XVIII are of interest; however their roles in the vascular healing process have not been fully elucidated [70]. 

Perlecan^−/−^ mice display severe phenotypic changes with most embryos dying prior to birth at E10-E12 as a result of ruptured basement membranes and consequential bleeding [71]. Cardiac tissue is especially affected. While basement membranes are present, they lack integrity and holes are observed in the myocardium. The few animals to survive until birth die soon after, suffering serious skeletal defects and defective cephalic development [72], emphasizing the importance of perlecan in a range of tissues. Focusing further on the cardiovascular consequences of perlecan deficiency, heterozygous mice show accelerated atherosclerosis in early lesions, implying a role in retention of lipoproteins [73]. Perlecan^−/−^ mice more seriously demonstrate a high incidence of arterial transposition, valve malformation and hyperplasia of SMC-specific α-actin-positive mesenchymal cells [74].

The modulation of vascular specific cells by perlecan has been clearly demonstrated; inhibiting smooth muscle cells while promoting endothelialization and displaying potent pro-angiogenic activity. The mechanism of these divergent effects is not yet fully understood. It was originally proposed that mouse derived perlecan interacted directly with endothelial cells through β_1_ and β_3_ integrins in a substantially RGD dependent manner [75]. However, later studies importantly differentiated that while mouse perlecan contains a prominent RGD motif, the human form contains no RGD at all [76]. An emerging explanation is that cell interactive effects of perlecan are mediated via heparin sulfate side chains sequestering growth factors such as fibroblast growth factor-2 (FGF-2), VEGF and hepatocyte growth factor (HGF). FGF-2 in particular readily binds to these side chains and can stimulate EC proliferation and migration [77].

The inhibition of vascular SMCs has been closely associated with perlecan expression levels in the cell [78]. High levels are found in quiescent phenotypes, with decreased amounts correlating to enhanced proliferation and in cases of vascular injury. As the healing response moves toward completion, perlecan accumulation in the cell induces a reduction in SMC proliferation via a PTEN signaling pathway [79]. This effect has been demonstrated *in vivo*, using a rat model undergoing balloon induced carotid artery injury. Specifically, expression and deposition of perlecan are low in the early stages following injury, while large amounts of perlecan remain deposited during later stages of lesion development, when SMC proliferation decreases. Up to 6 weeks after injury, perlecan remains abundant, suggesting that turnover is slow in the advanced lesion [80]. Mechanistically, the inhibition of SMCs by perlecan has been linked to the presence of functional heparin sulfate side chains. Mutant SMCs with abrogated side chain secretion show increased proliferation, consistent with the greater intimal hyperplasia observed in the arteries of mice also lacking HS side-chains. As with EC signaling, it is proposed that the effect of perlecan on SMCs is also mediated by the sequestering ability of the side-chains and specifically their binding of FGF-2 [81]. 

Hemostasis in normal vessels is mediated by a functional monolayer of endothelial cells, which express HSPGs that in turn bind and activate anti-thrombin III, inactivating thrombin [82], as well as inhibiting platelet adhesion and activation due to their highly negative charge [83]. Accordingly, perlecan has been shown to exert a direct influence on the clotting cascade, preventing thrombosis and inhibiting platelet adhesion. *In vitro*, platelets monitored using a quartz crystal microbalance adhered at similar levels to perlecan with and without HS side-chains, though aggregation was blocked only in the presence of HS chains only [84]. Endothelial cells expressing perlecan at normal levels were also shown to completely prevent occlusive thrombosis in a porcine carotid injury model. In contrast, perlecan-deficient cells had a 23% occlusion rate [32]. Notably, knockdown cells had a reduced ability to interact with FGF-2, a molecule frequently associated with perlecan activity.

The positive effects of perlecan shown *in vitro* have some evidence of translation to *in vivo* use. ePTFE vascular grafts dip-coated with perlecan were evaluated in a sheep carotid interposition model. After 6 weeks, perlecan-coated grafts showed a trend to less thrombus formation compared to uncoated controls and despite high inter-animal variability, a higher degree of endothelial cell coverage [84]. Additionally, a novel compound (RUS3108) that induces perlecan over-expression in SMCs was eluted from a stent platform in a rabbit iliac artery model. The cross-sectional area of in-stent neointima in the treated stent group was significantly reduced in comparison to bare stent or polymer coated alone controls [85].

### 2.4. Tropoelastin

Elastin is one of the major structural components of the vasculature and provides critical mechanical and biological properties [86]. Elastin is formed through the cross-linking of its soluble precursor tropoelastin, which is initiated through the action of the enzyme lysyl oxidase [87]. Tropoelastin is a 60 kDa highly extensible yet elastic monomer [88] that is cross-linked in the extracellular space and assembles to become the major component of elastic fibers [89]. 

The importance of normal elastin expression to the vasculature is evidenced by the severe disruptions observed in elastin knockout mice. Elastin^+/−^ animals present with hypertension, reduced vessel elasticity and display a reorganized arterial wall structure, but survive to normal age [90]. At birth, vascular function appears normal but progressive remodeling leads to reduced performance in adulthood. Approximately 60% of normal elastin levels are required for normal cardiac function, though this is achieved by significant vessel wall remodeling, notably with an increase in the number of elastic lamellae and layers of SMCs [91]. In elastin^−/−^ mice, arterial development appears normal until E17.5 when the internal elastic lamina (IEL) is severely compromised and subendothelial SMC proliferation becomes uncontrolled. Mice die prematurely of arterial obstruction [92]. 

The internal elastic lamina is particularly significant in providing signaling and support for luminal endothelial cells [93]. Commensurate with this role, various forms of elastin interact favorably with endothelial cells and support their growth. Elastin solubilized with oxalic acid (α-elastin) enhanced proliferation of ECs at low concentrations [94], while elastin peptides containing the GP/GVGAGVP or VGVAPG motifs are chemotactic for endothelial cells [95] and are consequently pro-angiogenic [96]. Fibrous scaffolds of bovine elastin were also shown to support endothelial cell growth up to 7 days in culture [97] however, the clearest enhancement of endothelialization has been demonstrated with recombinant human tropoelastin (rhTE). Attachment and proliferation of ECs was significantly increased in the presence of tropoelastin immobilized on a plasma deposited surface [15] and shown to be equivalent to fibronectin when coated on tissue culture plastic [98]. The formation of an EC monolayer was also encouraged on three-dimensional fibrous scaffolds containing tropoelastin, applied to the development of a vascular conduit [98]. Recently, it was shown that human coronary artery endothelial cell attachment to tropoelastin is in part mediated through the integrin a_v_β_3_, however the site of this interaction as well as other binding interactions has not been elucidated [99].

Elastin is also a potent autocrine regulator of SMC activity that inhibits proliferation and regulates migration. This effect is mediated directly by tropoelastin sequences via a repeating VGVAPG hexapeptide motif. Supply of this hexapeptide activates the same signaling cascade as the full-length protein in a specific and dose dependent manner [100]. *In vitro*, α-elastin has a strong dose dependent inhibitory effect on SMCs causing a greater than 50% reduction even at low concentrations (0.1 mg/mL) [94]. SMC inhibition has subsequently been demonstrated repeatedly for α-elastin scaffolds [101], hydrogels [102] and electrospun sheets [103]. Studies of SMCs derived from elastin^−/−^ mice provide the strongest evidence for the inhibitory effect of elastin. Cells lacking elastin were highly proliferative and increased by more than twofold by 72 hours, returning to wild type levels following the addition of recombinant tropoelastin to the media [104]. 

In addition, there is mounting evidence for the haemocompatibility of elastin and its derivatives and this subject has been reviewed in detail elsewhere [105]. In brief, elastin has demonstrated minimal platelet adhesion, degranulation and aggregation and was shown to be one of the least thrombogenic components of the vessel wall. In support of this finding, immobilized rhTE was found to have low thromobengicity on a plasma-activated steel surface [106], while porcine elastin-based scaffolds showed decreased thrombosis compared to commercial ePTFE in acute thrombogenicity tests in a carotid interposition model [107]. 

Translating the vascular compatibility of elastin-based materials *in vivo*, recombinant peptides based on the sequence of tropoelastin, have been shown to reduce thrombogenicity when used as surface coatings. Coated polyurethane catheters implanted in a rabbit model demonstrated decreased platelet deposition, reduced fibrin aggregation and ultimately improved patency [108]. Similar improvements in blood contacting properties were seen in a baboon arteriovenous shunt model using 4mm expanded polytetrafluoroethylene grafts coated with elastin-mimetic sequences [109]. Stents coated with an elastin sheet deployed in the coronary arteries of pigs have also demonstrated reduced neointimal hyperplasia compared to uncoated stents [110]. 

## 3. Conclusions and Future Prospects

Collectively, the outcomes from commercial graft and stent technologies have shown that simultaneously achieving reductions in neointimal hyperplasia, enhanced endothelialization and low thrombogenicity remains an elusive goal. This article sought to highlight the potential benefits of a biomimicry approach in the production of novel medical devices, employing biomolecules that have been shown to modulate multiple facets of vascular biology. A subset of candidates, chosen from the multitude found in the vasculature, standout as top tier candidates, and their favorable properties are summarized in Table 1.

**Table 1:** Summary of vascular biological effects of biomolecule candidates.

Candidate	Effect on ECs	Effect on SMCs	Blood Compatibility	Translation to Date
**Fibrillin-1**	Proliferation, *enhanced*	Proliferation, *inhibited*	Not yet tested	Enhanced fibroblast attachment to PU scaffold
**Fibulin-5**	Attachment *enhanced*; *apoptosis reduced*	Proliferation, *inhibited*	Not yet tested	No translation
**Tropoelastin**	Attachment, proliferation, *enhanced*	Proliferation, *inhibited*	Hemocompatible; minimal activation of platelets	Improves EC binding, growth on steel; reduces thrombogenicity of catheters, ePTFE grafts
**Perlecan**	Proliferation, *enhanced*	Proliferation, hyperplasia (rat model) *inhibited*	Direct inhibition of thrombosis	Less thrombus present on ePTFE grafts, greater ECs; Stents show less neointima

With a growing appreciation of the need for biointegration of vascular devices, it is anticipated that these candidates will increasingly be translated to metal and polymeric substrates for *in vivo* evaluation. This translation will be achieved more quickly in combination with emerging coating technologies that facilitate binding of biomolecules, in a lasting yet bioactive manner. Recent improvements, set apart from conventional plasma polymerisation, eliminating the need for chemical linkers is one such example [111]. Future developments will continue to aim to produce a novel device platform that can achieve controlled integration with the vasculature through multifaceted biomimicry. Ultimately, the goal remains the development of truly biocompatible materials for a wide range of vascular applications, including stents and graft materials, thereby meeting a large unmet need in cardiovascular medicine.
